# A method for rapid similarity analysis of RNA secondary structures

**DOI:** 10.1186/1471-2105-7-493

**Published:** 2006-11-08

**Authors:** Na Liu, Tianming Wang

**Affiliations:** 1 Department of Applied Mathematics, Dalian University of Technology, Dalian 116024, China; 2 College of Advanced Science and Technology, Dalian University of Technology, Dalian 116024, China; 3 Department of Mathematics, Hainan Normal University, Haikou 571158, China

## Abstract

**Background:**

Owing to the rapid expansion of RNA structure databases in recent years, efficient methods for structure comparison are in demand for function prediction and evolutionary analysis. Usually, the similarity of RNA secondary structures is evaluated based on tree models and dynamic programming algorithms. We present here a new method for the similarity analysis of RNA secondary structures.

**Results:**

Three sets of real data have been used as input for the example applications. Set I includes the  structures from *5S rRNAs. *Set II includes the secondary  structures from *RNase P* and *RNase MRP*. Set III includes the structures from *16S rRNAs*. Reasonable phylogenetic trees are derived for these three sets of data by using our method. Moreover, our program runs faster as compared to some existing ones.

**Conclusion:**

The famous Lempel-Ziv algorithm can efficiently extract the information on repeated patterns encoded in RNA secondary structures and makes our method an alternative to analyze the similarity of RNA secondary structures. This method will also be useful to researchers who are interested in evolutionary analysis.

## Background

RNA secondary structures play an important role in determining the functions of RNA molecules. Some of them have been accepted as good data for evolutionary analysis. With the completion of the sequencing of the genomes of human and other species, major structural biology resources have been harnessed to predict functions. More and more RNA structures are accumulated and we know little about their functions. This calls for the development of cost-effective computational methods to predict RNA functions, which will provide preliminary information for biologists and guide biological experiments. Earlier studies usually adopt dynamic programming algorithms and tree models. Shapiro et al [1] proposed to compare RNA secondary structures by using tree models. Hofacker et al [2] compared RNA secondary structures by aligning the corresponding base pairing probability matrices that were computed by McCaskill's partition function algorithm [3]. Because these methods rely on dynamic programming algorithms, they are compute-intensive. Constructing tree models is based on the idea that the stems or helices dominantly stabilize the secondary structures. So they ignore their primary sequences and focus on so-called elementary units (stem and loop, etc) for the similarity analysis. There are other works, in which tree models were constructed to analyze the similarity of RNA secondary structures [4-8]. Recently Liao et al [9] have proposed to use graphs to represent RNA secondary structures and then derive some invariants from graphs to compare RNA secondary structures. This idea is from the study of DNA sequences [10-13]. It has been stated [10] that invariants actually reflect some characterizations of biological structures or sequences and may be regarded as indicators. Some information will be lost, however, and how to obtain and select suitable invariants to characterize biological sequences so as to compare DNA sequences effectively is still unsolved. What's more, the graphical representations don't work well when the size of the RNA secondary structure is large. Obviously, for complex RNA secondary structures, more information is lost, which will affect the similarity analysis. Popular tools for optimal alignment of RNA secondary structures include RNAdistance [1], RNAforester [14] etc. RNAdistance uses the tree models to coarsely represent RNA secondary structures, and compares RNA secondary structures based on tree edit distance measure. RNAforester supports the computation of pairwise and multiple alignment of structures based on tree alignment measure.

In this paper we propose a novel method for the similarity analysis of RNA secondary structures, where pseudoknots are also taken into account. In our approach, each secondary structure is transformed into a linear sequence. The linear sequence not only contains the information on the corresponding RNA primary structure, but also contains the information on the base pairing.

Furthermore, standard and famous Lempel-Ziv algorithm [15] is employed for the similarity analysis. Of course, we have tested the validity of our method by analyzing three sets of real data. The results obtained by our method are comparable to those given by other authoritative methods. What's more, the whole process is easy to operate. It can yield results rapidly.

## Results

### Materials

Three sets of real data are used to test our method. RNA secondary structures in set II are from *RNase P *and *RNase MRP*. They are distantly related and there is little sequence homology between them. These secondary structures are used to test distant RNA secondary structures. They are mainly obtained from the *RNase P *Database [16] and the remaining secondary structures are obtained from [17]. The names of the RNA secondary structures from *RNase P *are: *Synechocystis sp.PCC6803, Anacystis nidulans PCC6301, Pseudoanabaena sp.PCC6903, Anabaena sp.PCC7120, Porphyra purpurea chloroplast, Thermotoga maritima, Agrobacterium tumefaciens, Rhodospirillum rubrum, Bacillus subtilis, Reclinomonas americana mitochondria, Sulfolobus acidocaldarius, Methanococcus jannaschii, Halobacterium cutirubrum, Human (nuclear) P*. The RNA secondary structures from *RNase MRP *are obtained from [17], whose names are: *Human, Bovine, Mouse, Rat*. RNA secondary structures in set I are from *5S rRNAs*. They are provided by Maciej Szymanski, who has developed the 5S Ribosomal RNA Database [18]. The names of the *5S rRNAs *used in our study are *Halobacterium spl, Pyrodictium occultum, Sulfolobus spl, Actinia equina, Dicyema misakiense, Basidiobolus magnus, Chrysaora quinque, Christiansenis pallida *and *Planocera recticulata*. RNA secondary structures in set III are from *16S rRNAs*. The names of the *16S rRNAs *are *Thermoproteus tenax, Halobacterium, Bacteoides, Bacillus, Mus musculus, Synechococcus, Thermotoga, Saccharomyces cerevisiae, Homo sapiens, Escherichia coli, Methanococus vannielli, Thermococcus celer, Vairimorpha *and *Methanobacterium*.

### The similarity analysis of set I and set II by using our method

**Figure 1:**
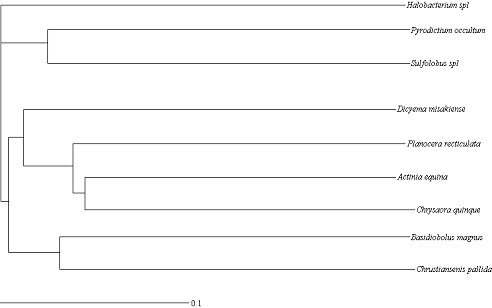
**Neighbor-joining tree for the data in set I**. It is obtained by our method and drawn by Treeview program.

**Figure 2:**
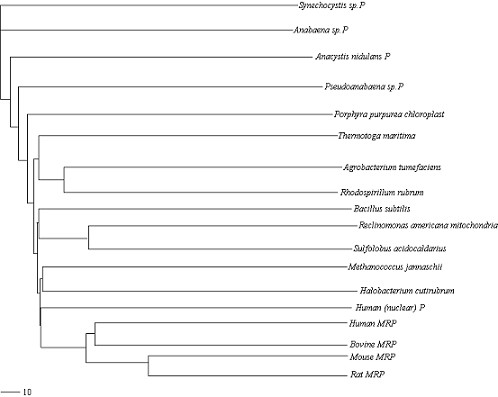
**Neighbor-joining tree for the data in set II**. It is obtained by our method and drawn by Treeview program.

The goal of our study is to compare RNA secondary structures and analyze their similarity. Given a set of RNA secondary structures, our method requires the following main operations for the similarity analysis: Firstly the non-linear complex RNA secondary structures are transformed into linear characteristic sequences. Secondly, these linear sequences are decomposed according to the rule of Lempel-Ziv algorithm to evaluate the LZ complexity. Thirdly, the similarity degree between any two structures is measured by our distance formula, as shown in Method section. Lastly, by arranging all the values into a matrix, we obtain a pair-wise distance matrix. It contains the information on the similarity of this set of RNA secondary structures. We have used our method to analyze the similarity of set I and set II, respectively. Its validity may be better reflected by its application to reconstruct phylogenetic trees. Hence, for the two sets of data, we input their pair-wise distance matrices, obtained by our methods, into the Neighbor program in the Phylip package [19], respectively. By choosing Neighbor-joining option, we obtain two phylogenetic trees for the two sets, which are drawn by Treeview program [20] and are shown in Figure 1 and Figure 2.

## Discussion

Lempel-Ziv algorithm is an algorithm that is related to minimal length encoding. Its successful application to the evolutionary analysis of DNA sequences has indicated that Lempel-Ziv algorithm is an alternative to the similarity analysis of biological sequences. To our knowledge, the concept of applying Lempel-Ziv algorithm to the similarity analysis of RNA secondary structures hasn't been adopted by any other researcher. The introduction of our method in Method section indicates that this is a relatively simple and rapid method for the similarity analysis of RNA secondary structures. We owe the efficiency of this method mainly to the Lemple-Ziv algorithm, which can effectively extract the repeated patterns encoded in linear sequences.

**Figure 3:**
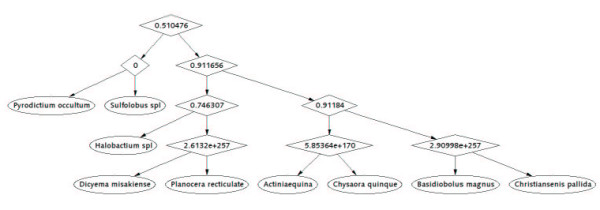
**Cluster tree for the data in set I**. It is obtained by using RNAforester program. The tree is derived based on the similarity scores between any pair of RNA forests.

**Figure 4:**
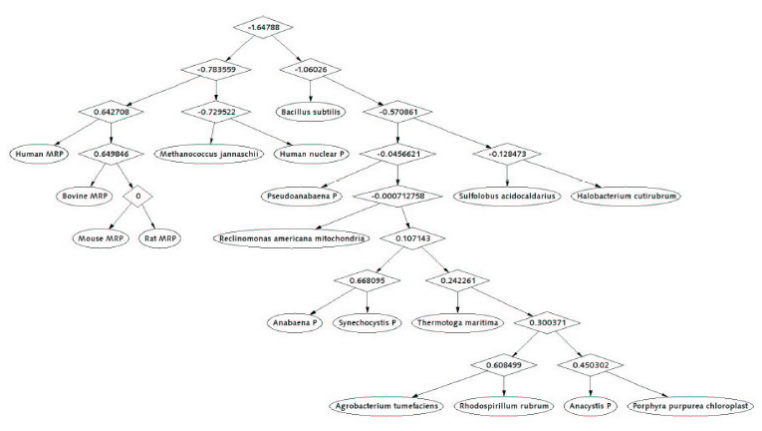
**Cluster tree for the data in set II**. It is obtained by using RNAforester program. The tree is derived based on the similarity scores between any pair of RNA forests.

For comparison, we employ RNAforester program to perform the similarity analysis on the same data. This program calculates the similarity score for any pair of RNA secondary structures under the proposed scoring scheme. The similarity relationship is displayed in a cluster tree. By performing the RNAforester program on set I and set II, we obtain two cluster trees, as shown in Figure 3 and Figure 4. The numbers in the interior nodes of the cluster trees usually represent the similarity scores between the two sub-clusters that the interior nodes connect, respectively. Note that we set 0.7 as the clustering threshold when we run RNAforester program. Thus the similarity score that is not less than 0.7 will be replaced by 0 in the cluster tree. The efficiency of RNAforester program in analyzing the data from set I and set II is evaluated by Figure 3 and Figure 4.

At first, we compare Figure 1 with Figure 3. From Figure 1, we observe that: 1. *Actinia equina, Chrysaora quinque and Planocera recticulata *are grouped closely (they belong to *Animalia*); 2. *Basidiobolus magnus *and *Christiansenis pallida *are grouped closely (they belong to *fungi*); 3. *Pyrodictium occultum, Halobacterium spl *and *Sulfolobus spl *(they belong to *Archaebacteria*) are clearly separated from the rest; 4. *Dicyema misakiense *is placed closer to *Animalia *than to *fungi *(it belongs to *mesozoa*). The relationship described by our method is in accordance with the one described in [21,22]. In contrast to Figure 1, we find in Figure 3, obtained by using RNAforester program, that *Halobacterium spl *is separated from the cluster that *Pyrodictium occultum *and *Sulfolobus spl *belong to. Obviously this is not reasonable. Then we compare Figure 2 with Figure 4. From Figure 2, we observe that our result is consistent with the theory that is suggested in [23-26]: MRP evolved from a Eukaryotic Nuclear P in the nucleus of an early Eukaryote. Figure 2 indicates that mrpRNA are more similar to eukaryotic pRNA than to prokaryotic pRNA. Furthermore, *Synechocystis sp.PCC6803*, *Anacystis nidulans PCC6301*, *Pseudoanabaena sp.PCC6903*, *Anabaena sp.PCC7120 *and *Porphyra purpurea chloroplast *are grouped closely, named cluster I for convenience; *Thermotoga maritima, Agrobacterium tumefaciens *and *Rhodospirillum rubrum *are grouped closely, named cluster II. Cluster I and cluster II are adjacent. In Figure 4, *Halobacterium cutirubrum *is put far away from *Methanococcus jannaschii*. Furthermore, *Anacystis nidulans P *is separated far from *Synechocystis sp.P *and *Anabaena sp.P. Bacillus subtilis *and *Reclinomonas americana mitochondria *aren't placed closely. This conformation doesn't accord with the one demonstrated by Collins et al.

In general, our method can compare secondary structures reasonably, with the results consistent with those from [23-26]. For the two data sets, our algorithm performs better than RNAforester program. Additionally, our analysis results favor the proposal that RNA secondary structures are useful materials for evolutionary analysis.

**Figure 5:**
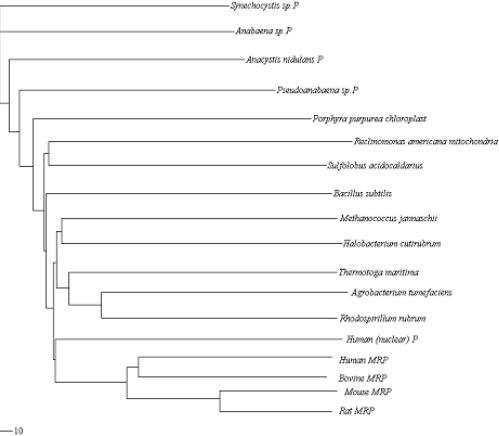
**Neighbor-joining tree for the data in set II**. It is obtained by performing LZ algorithm on RNA primary structures, i.e. the step to extract linear characteristic sequences from RNA secondary structures has been ignored.

It seems that our method is heavily biased towards comparing sequences, not secondary structures. However, in fact, this is not the truth. We now apply Lempel-Ziv algorithm directly to RNA sequences to see whether the result obtained by this method is better than ours. As a result, the phylogenetic tree for the data in set II has much divergence from ours, shown in Figure 5 (drawn by Treeview).

It's obvious that there exists unreasonable topology that depicts the similarity relationship of these RNA secondary structures in Figure 5. For example, *Thermotoga maritima, Agrobacterium tumefaciens *and *Rhodospirillum rubrum *are placed close to the RNase MRP RNAs and are separated far away from the branch for *Synechocystis sp.P *and *Anabaena sp.P*, etc, which simultaneously leads to the separation of *Sulfolobus acidocaldarius *from *Methanococcus jannaschii *and *Halobacterium cutirubrum*. In nature, *Thermotoga maritima, Agrobacterium tumefaciens *and *Rhodospirillum rubrum *belong to Eubacterial RNase P and should be grouped close to *Synechocystis sp.P *and *Anabaena sp.P*, etc. Figure 5 has favored our claim, i.e. our characteristic sequences do grasp some information on RNA secondary structures (base pairing).

The introduction of the Lempel-Ziv algorithm to the similarity analysis makes our algorithm run fast. Table 1 lists the general time and space complexity of our method and RNAforester program. In Table 1, the relationship between the size (length) of RNA secondary structure and the time complexities hasn't been indicated explicitly for the RNAforester program. We may make approximate estimation. In theory, the total number of the nodes of an RNA forest scales linearly with the size of the RNA secondary structure. For RNA secondary structures that exist in nature, the maximum length of an unpaired region and the branching degree can be considered to be bounded by some constants, which determines that the degree of an RNA forest is expected to stay a constant. Hence the running time *O*(|*F*_1_||*F*_2_|*deg*(*F*_1_))*deg*(*F*_2_))[27] is equivalent to *O*(*n*_1_*n*_2_), where *n*_1_*n*_2 _is the product of the sizes of the two RNA secondary structures being compared.

**Table 1:** Time/Space complexities of our method and the RNAforester program.

Algorithm Name	Running Time	Space requirement	Reference
^*a*^*RNA forester*	O(|*F*_1_||*F*_2_|*deg*(*F*_1_)*deg*(*F*_2_))	O(|*F*_1_||*F*_2_|*max*(*deg*(*F*_1_), *deg*(*F*_2_)))	[27]
^*b*^*Our method*	O(*N*^2^)	O(*N*^2^)	

^*a*^|*F*_*i*_| is the number of nodes in the forest *F*_*i *_and *deg*(*F*_*i*_) is the degree of *F*_*i*_; ^*b*^*N *is the average size.

On the other hand, we have compared the execution time of our method with that of RNAforester by using some RNA secondary structures of various sizes. The results are listed in Table 2. It's obvious that our algorithm performs faster.

**Table 2:** Execution times required by our algorithm and the RNAforester program.

Species Name	Execution Time required by RNAforester	Execution Time required by our method
^*c*^*Two *5*S rRNAs*	12.62 s	1.52 s
^*d*^*Two RNase P RNAs*	35.36 s	6.96 s
^*e*^*Two *16*S rRNAs*	1583.12 s	176.68 s

The two algorithms have been performed on the same representative RNAs for comparison. Letter s represents seconds. In ^*c*^, *Halobacterium spl *and *Christiansenis pallida *are chosen to compare. In ^*d*^, *Porphyra purpurea chloroplast *and *Bacillus subtilis *are chosen to compare. In ^*e*^, *Thermotoga *and *Saccharomyces cerevisiae *are chosen to compare.

**Figure 6:**
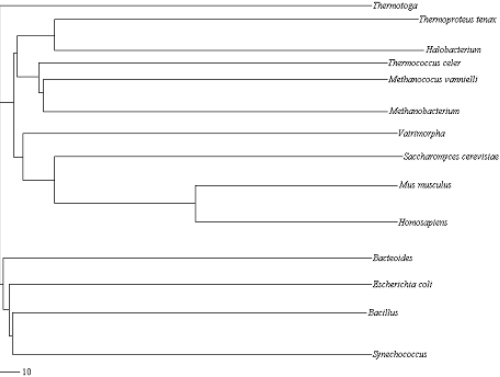
**Neighbor-joining tree for the data in set III**. It is obtained by our method and drawn by Treeview program.

Additionally, we have performed our program on a set of *16S rRNAs*, whose secondary structures are more complex and the sizes of which are relatively larger. The result is shown in Figure 6, drawn by Treeview. Their similarity relationship has been reasonably derived by our method. *Thermoproteus tenax, Halobacterium, Methanococus vannielli, Thermococcus celer *and *Methanobacterium *have been clustered together, which is consistent with the fact that they are of *Archaea*. *Mus musculus, Saccharomyces cerevisiae, Homo sapiens, Vairimorpha *are clustered together, which is consistent with the fact that they are of *Eucaya*. The left are of *Bacteria*.

## Conclusion

Here we have proposed a new method to analyze the similarity of RNA secondary structures (pseudoknots are taken into account). It is a simple method that yields results reasonably and rapidly. Our algorithm is not necessarily an improvement as compared to some existing methods, but an alternative for the similarity analysis of RNA secondary structures. The new method doesn't require sequence alignment and the construction of tree models. It is based on linear characteristic sequences that we define for RNA secondary structures and the famous Lempel-Ziv algorithm that relates to minimal length encoding. The characteristic sequences contain the information from RNA primary structures and the base pairs formed in RNA secondary structures. The Lempel-Ziv algorithm effectively extracts the information on the repeated patterns encoded in long sequences. The example applications of our method to three sets of real data and its comparison with other methods verify the validity of our method. From the comparisons, we conclude that our method performs well on distantly related RNA secondary structures. In our approach, complicated computation is avoided. The whole process is easy to operate. What's more, the size of RNA secondary structure is not problematic.

Of course, there is defect in our approach: when non-linear RNA secondary structures are transformed into linear characteristic sequences, some information may be lost. However, our test has indicated that our method can yield results reasonably, i.e. our method can extract some key information from RNA secondary structures.

## Methods

### Lempel-Ziv algorithm and LZ complexity

Let *S*, *Q *and *R *be sequences over a finite alphabet Λ, *l*(*S*) be the length of *S, S*(*i*) be the *ith *element of *S *and *S*(*i,j*) be the subsequence of *S *that starts at position *i *and ends at position *j*. Note that *S*(*i,j*) = ∅, for *i *> *j*. The contatenation of *Q *and *R *forms a new sequence *S *= *QR*, where *Q *is called a prefix of *S*, and *S *is called an extension of *Q *if there exists an integer *i *such that *Q *= *S*(1, *i*).

An extension *S *= *QR *of *Q *is reproducible from *Q *denoted by *Q *→ *S*, if there exists an integer *p *≤ *l*(*Q*) such that *R*(*k*) = *S*(*p*+*k*-1), for *k *= 1,2,.., *l*(*R*). For example: *AACUT *→ *AACUTACU *with *p *= 2. A non-null sequence *S *is producible from its prefix *S*(1, *j*), denoted by *S*(1, *j*) ⇒ *S*, if *S*(1, *j*) → *S*(1, *l*(*S*) - 1). For example: *CCUA *⇒ *CCU AU AUT *with *p *= 3.

The difference between producibility and reproducibility is that the former allows for an extra "different" symbol at the end of the extension process which is not permitted in the latter. Therefore an extension which is reproducible is always producible but the reverse may not always be true.

Any non-null sequence *S *can be built from a production process by iterative self-deleting-building process where at the *ith *step *S*(1, *h*_*i*-1_) ⇒ *S*(1, *h*_*i*_), ∅ = *S*(1, 0) ⇒ *S*(1, 1). An m-step production process of *S *leads to a parsing of *S *into *H*(*S*) = *S*(1, *h*_1_) • *S*(*h*_1 _+ 1, *h*_2_) •......• *S*(*h*_*m*-1 _+ 1, *h*_*m*_), which is called the history of *S*, and *H*_*i*_(*S*)= *S*(*h*_*i*-1 _+ 1, *h*_*i*_) is called the *ith *component of *H*(*S*).

A component *H*_*i*_(*S*) and the corresponding production step *S*(1, *h*_*i*-1_) ⇒ *S*(1, *h*_*i*_) are called exhaustive if *S*(1, *h*_*i*-1_) → *S*(1, *h*_*i*_) is not true. A history is called exhaustive if each of its components (with a possible exception of the last one) is exhaustive. What's more important, the exhaustive history of any non-null sequence is unique. For example, for the sequence *S *= *UUCGAGGUCGGA*, its exhaustive history is *EH*(*S*)= *U*•*UC*•*G*•*A*•*GG*•*UCGG*•*A*.

Let *c*(*S*) be the number of components in the exhaustive history of *S*. It is the least possible number of steps needed to generate *S *according to the whole Lempel-Ziv algorithm, so *c*(*S*) becomes an important complexity indicator.

### Linear characteristic sequences of RNA secondary structures

**Figure 7:**
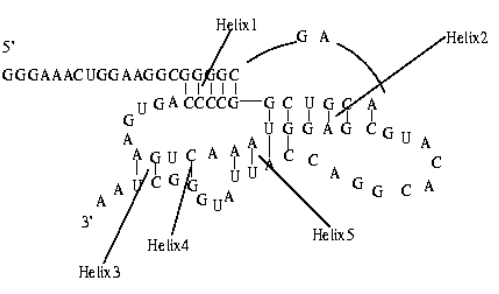
**A simulated secondary structure**. It contains a pseudoknot. And five Helices are formed according to our definition.

Usually, A, C, G, U are used to denote the four bases(nucleotides) in RNA sequences (primary structures). An RNA sequence can thus be represented by *R *= *r*_1_*r*_2_.....*r*_*n*_, where *r*_*i *_is called the *i*^*th*^(ribo)nucleotide. Each *r*_*i *_belongs to the alphabet {A, C, G, U}. The secondary structure of an RNA molecule is the collection of base pairs that occur in its 3D structure. For each secondary structure, there are two terminals: 5'-terminal and 3'-terminal. Figure 7 shows a simulated RNA secondary structure. Its RNA sequence (from 5'-terminal to 3'-terminal)is:

*GGGAAACUGGAAGGCGGGGCGAACGUCGGCCCCAGUGAAGUCAAAUGGAGCGUACACGGACCAUUAUGGGCUAA*.

In this section, we will define linear characteristic sequences for RNA secondary structures. In other words, we will transform non-linear RNA secondary structures into linear sequences. In our research, a group of consecutive base pairs (including one base pair) is called a helix and *i *is called the position of nucleotide *r*_*i*_. The open regions surrounded by single stranded bases are called loops. Each helix is numbered from the 5'-terminal to 3'-terminal: The first helix is called Helix1, and the second helix is called Helix2,......etc. The rule for transformation is as such: For an RNA secondary structure S with N helices, write its RNA sequence with the letters in Helix1 upper case and the rest small case; then write in succession its RNA sequence with the letters in Helix2 upper case and the rest small case;.......then write in succession its RNA sequence with the letters in HelixN upper case and the rest small case. Now a linear sequence has been obtained by following the above-mentioned rule. We call it linear characteristic sequence of *S*, abbreviated to L(S). Take the simulated secondary structure for example, it has five Helices.

According to our rule, the linear characteristic sequence of the simulated secondary structure is as follows:

gggaaacuggaaggcGGGGCgaacgucgGCCCCagugaagucaaauggagcguacacggaccauuaugggcuaagggaaacuggaaggcggggcgaACGUCGgccccagugaagucaaaUGGAGCguacacggaccauuaugggcuaagggaaa

cuggaaggcggggcgaacgucggccccagugaAGucaaauggagcguacacggaccauuaugggCUaagggaaacuggaa

ggcggggcgaacgucggccccagugaaguCaaauggagcguacacggaccauuaugGgcuaagggaaacuggaaggcgggg

cgaacgucggccccagugaagucaAAUGgagcguacacggacCAUUaugggcuaa

which is obtained by adjoining the following five sequences in succession.

Helix1: *gggaaacuggaaggcGGGGCgaacgucgGCCCCagugaagucaaauggagcguacacggaccauuaugggcuaa*

Helix2: *gggaaacuggaaggcggggcgaACGUCGgccccagugaagucaaaUGGAGCguacacggaccauuaugggcuaa*

Helix3: *gggaaacuggaaggcggggcgaacgucggccccagugaAGucaaauggagcguacacggaccauuaugggCUaa*

Helix4: *gggaaacuggaaggcggggcgaacgucggccccagugaaguCaaauggagcguacacggaccauuaugGgcuaa*

Helix5: *gggaaacuggaaggcggggcgaacgucggccccagugaagucaAAUGgagcguacacggacCAUUaugggcuaa*

### Distance computation and pair-wise distance matrix

Lempel et al have proposed that, for any given sequences *Q *and *S*, *c*(*QS*) ≤ *c*(*Q*)*+ c*(*S*) always remains valid. This formula shows that the steps required to extend *Q *to *QS *are always less than the steps required to build *S *from ∅. Recently, Otu et al [28] concluded that the more similar the sequence *S *is to sequence *Q*, the smaller *c*(*QS*) - *c*(*Q*) is. That is *c*(*QS*) - *c*(*Q*) depends on how much *S *is similar to *Q*.

For example, let *Q*, *S*, *R *represent three short RNA sequences defined over the alphabet {*A*, *C*, *G*, *U*}, where *S *= *UUACGUAAUGU,Q *= *AGUCCCUAGGA, R *= *UACCGAUAAG*. By the rule mentioned above, the corresponding exhaustive histories of *S*, *Q*, *R*, *SR*, *QR*, *SQ *are: *EH*(*S*) = *U*•*UA*•*C*•*G*•*UAA*•*UG*•*U, EH*(*Q*) = *A*•*G*•*U*•*C*•*CCU*•*AGG*•*A, EH*(*R*) = *U*•*A*•*C*•*CG*•*AU*•*AA*•*G, EH*(*SR*) = *U*•*UA*•*C*•*G*•*UAA*•*UG*•*UUACC*•*GA*•*UAAG, EH*(*QR*) = *A*•*G*•*U*•*C*•*CCU*•*AGG*•*AU*•*AC*•*CG*•*AUAA*•*G, EH*(*SQ*) = *U*•*UA*•*C*•*G*•*UAA*•*UG*•*UAG*•*UC*•*CC*•*UAGG*•*A*. We can find that we need 2 steps to build *R *from *S*, 4 steps to build *R *from *Q*, 4 steps to build *Q *from *S*. So we say *R *is more similar to *S *than to *Q*. The reason is that *S *and *R *share the common patterns *UAC *and *UAA*.

Based on this hypothesis, Otu et al have used the Lempel-Ziv algorithm to successfully construct phylogenetic trees from DNA sequences, which verifies the efficiency of Lempel-Ziv algorithm in analyzing the similarity of linear biological sequences.

Therefore we adopt the following formula to evaluate the distance between secondary structures S and Q, which is slightly different from [28]:

Rd(S,Q)={c(L(S)L(Q))−c(L(S))+c(L(Q)L(S))−c(L(Q))c(L(S)L(Q))+c(L(Q)L(S))S≠Q0,S=Q
 MathType@MTEF@5@5@+=feaafiart1ev1aaatCvAUfKttLearuWrP9MDH5MBPbIqV92AaeXatLxBI9gBaebbnrfifHhDYfgasaacH8akY=wiFfYdH8Gipec8Eeeu0xXdbba9frFj0=OqFfea0dXdd9vqai=hGuQ8kuc9pgc9s8qqaq=dirpe0xb9q8qiLsFr0=vr0=vr0dc8meaabaqaciaacaGaaeqabaqabeGadaaakeaacqWGsbGucqWGKbazcqGGOaakcqWGtbWucqGGSaalcqWGrbqucqGGPaqkcqGH9aqpdaGabeqaauaabeqaciaaaeaadaWcaaqaaiabdogaJjabcIcaOiabdYeamjabcIcaOiabdofatjabcMcaPiabdYeamjabcIcaOiabdgfarjabcMcaPiabcMcaPiabgkHiTiabdogaJjabcIcaOiabdYeamjabcIcaOiabdofatjabcMcaPiabcMcaPiabgUcaRiabdogaJjabcIcaOiabdYeamjabcIcaOiabdgfarjabcMcaPiabdYeamjabcIcaOiabdofatjabcMcaPiabcMcaPiabgkHiTiabdogaJjabcIcaOiabdYeamjabcIcaOiabdgfarjabcMcaPiabcMcaPaqaaiabdogaJjabcIcaOiabdYeamjabcIcaOiabdofatjabcMcaPiabdYeamjabcIcaOiabdgfarjabcMcaPiabcMcaPiabgUcaRiabdogaJjabcIcaOiabdYeamjabcIcaOiabdgfarjabcMcaPiabdYeamjabcIcaOiabdofatjabcMcaPiabcMcaPaaaaeaacqWGtbWucqGHGjsUcqWGrbquaeaacqaIWaamcqGGSaalaeaacqWGtbWucqGH9aqpcqWGrbquaaaacaGL7baaaaa@7D4A@

The denominator in [28] is equivalent to [*c*(*L*(*S*)*L*(*Q*))+*c*(*L*(*S*)*L*(*Q*))]/2, which leads to the fact that the distance calculated by the formula proposed in [28] will always be twice as much as the distance calculated by our formula. As you know, a constant will not affect the similarity analysis at all. We choose to use the formula mentioned above mainly because its expression is simpler. The formula in [28] has been proven to be a distance metric by Out et al. Thus *Rd*(*S, Q*) also satisfies the conditions required by a distance metric.

It's obvious that the more similar S is to Q, the smaller *c*(*L*(*S*)*L*(*Q*))-*c*(*L*(*S*)) and *c*(*L*(*Q*)*L*(*S*))-*c*(*L*(*Q*)) are, and then the smaller *Rd*(*S,Q*) is.

Generally, given *n *RNA secondary structures *S*_1_, *S*_2_,......, *S*_*n*_, we can obtain their linear characteristic sequences by the above-mentioned rule, which are *L*(*S*_1_), *L*(*S*_2_),......, *L*(*S*_*n*_). They are linear sequences defined over alphabet {A, C, G, U, a, c, g, u,} and carry the information on RNA secondary structures. Then, by using Lempel-Ziv algorithm, the distance between any pair of structures, *Rd*(*S*_*i*_, *S*_*j*_), may be rapidly computed. By arranging them into a matrix, a pair-wise distance matrix is obtained, denoted by *RD*. *RD *=(*Rd*(*S*_*i*_, *S*_*j*_)) contains the information on the similarity/dissimilarity between any pair of RNA secondary structures.

Based on what Otu et al have proven, we can easily conclude that our distance metric is also valid for inferring phylogeny of species because it satisfies all the conditions for phylogeny analysis. Hence our pair-wise distance matrix may be input the programmes for phylogeny inference to study the phylogeny for RNA molecules based on their secondary structures.

## Authors' contributions

NL conceived of the study and drafted the manuscript. NL participated in the design of the algorithm. NL and TMW performed the comparison of RNA secondary structures and tested the algorithm.
